# Dying to cooperate: the role of environmental harshness in human collaboration

**DOI:** 10.1093/beheco/arab125

**Published:** 2021-11-12

**Authors:** Paul Ibbotson, Cristian Jimenez-Romero, Karen M Page

**Affiliations:** Faculty of Wellbeing, Education & Language Studies, Open University, Walton Hall, Milton Keynes MK7 6AA, UK; Faculty of Wellbeing, Education & Language Studies, Open University, Walton Hall, Milton Keynes MK7 6AA, UK; Department of Mathematics, University College London, Gower Street, London WC1E 6BT,UK

**Keywords:** communication, cooperation, cost of living, environmental stress, resource accumulation, social foraging strategies

## Abstract

It has been proposed that environmental stress acted as a selection pressure on the evolution of human cooperation. Through agent-based evolutionary modelling, mathematical analysis, and human experimental data we illuminate the mechanisms by which the environment influences cooperative success and decision making in a Stag Hunt game. The modelling and mathematical results show that only cooperative foraging phenotypes survive the harshest of environments but pay a penalty for miscoordination in favourable environments. When agents are allowed to coordinate their hunting intentions by communicating, cooperative phenotypes outcompete those who pursue individual strategies in almost all environmental and payoff scenarios examined. Data from human participants show flexible decision-making in face of cooperative uncertainty, favouring high-risk, high-reward strategy when environments are harsher and starvation is imminent. Converging lines of evidence from the three approaches indicate a significant role for environmental variability in human cooperative dynamics and the species-unique cognition designed to support it.

## Teaser

It has been proposed that harsh environments can apply pressure on organisms to evolve cooperative behaviors. We find that cooperation is the only strategy that survives the harshest of environments and that humans opt for riskier, more cooperative strategies when their energy supply is depleted. Together the results show the significant role environmental harshness plays in human cooperative dynamics and the species-unique cognition designed to support it.

## INTRODUCTION

There have been numerous descriptions of the possible climatic and ecological drivers of hominin evolution (Darwin 1871; DeMenocal 1995; Potts 1998; Sepulchre et al. 2006; Andras et al. 2007; Richerson et al. 2008; Carto et al. 2009). Because of humans’ status in the natural world as ultra-co-operators, of particular interest is the role that adverse Plio-Pleistocene ecology played in providing a “common enemy” for cooperative phenotype selection (Kropotkin 1902; Gibbons and Dugatkin 1992; Kümmerli et al. 2009; Tomasello et al. 2012; Tomasello and Vaish 2013). This paper reports novel insights into the relevant mechanisms driving cooperative success and decision making by combining three approaches. First, we implement the Stag Hunt Game (SHG) as a model of human cooperation, using agent-based modelling (ABM) to explore some of the dynamics of collaboration played out against various environmental backdrops. Second, we draw general formal conclusions about the fitness of different cooperative phenotypes across a range of environmental conditions using mathematical analysis. Finally, we test some of the implications of these models in an experiment where human participants make strategic decisions in real-time about whether to cooperate.

The rationale for combining these approaches is that, individually, they are best suited to answering slightly different questions about the same underlying phenomena, but when combined, their complementary strengths amount to a more persuasive argument, especially when different lines of evidence converge on the same interpretation. For example, a strength of the ABM approach allows us to examine emergent properties of complex systems with many variables (a population of cooperative agents) that would be mathematically cumbersome if not intractable to analyze a priori. ABMs simulate the behavior of the system’s constituent units (the agents) and their interactions, capturing emergence from the bottom up. Thus, the ABM provides a very natural framework for describing and simulating behavior at a systems level that cannot be reduced to the system’s parts. Having established what the relevant emergent properties are as a result of ABM output, we simplify and generalize these properties to gain a deeper understanding of their dynamics through mathematical analysis. Specifically, we divided Stag and Hare hunters into well-fed and poorly-fed individuals, which combine in heterotypic and homotypic pairs and analyse the environmental threshold at which they can survive. Finally, the human experiment investigates whether human decision making reflects the optimal strategies for the specific environmental harshness observed in the models.

We use the SHG as a model of real-life cooperation (Skyrms 2003; Tomasello et al. 2012) rather than the more widely-studied Prisoner Dilemma Game (PDG) as the argument has been strongly made that PDGs do not reflect the reality of many socio-ecological contexts (Clements and Stephens 1995; Maynard Smith and Szathmary 1995; Dugatkin 1997; Alvard 2001) nor do they capture the collective-action problems many animals face (Runge 1984; Ostrom 1990; Hirshleifer 1999; Bardhan 2000). In the SHG, the payoff of collaboration – hunting a stag – is greater than going it alone and hunting a hare, classically by a 2:1 ratio. To collaborate one must give up the low-risk low-payoff hare to obtain the high-risk high-payoff stag. “High-risk” because if a Stag-hunter cannot coordinate their intentions with that of a like-mined partner, they waste time and energy on a venture with no prospect of rewards and end up worse off than hunting a Hare alone. Crucially, the co-operator in this game is not under the same temptation to defect as they are in the Prisoner’s Dilemma Game (PDG) as what is good for them (a Stag) is also good for their partner (Nowak and Sigmund 1992; Cooper et al. 1996; Epstein 1998; Sherratt and Roberts 1998; Koella 2000; Andras et al. 2007; Nowak et al. 2010; Press and Dyson 2012; Smaldino et al. 2013; Cardinot et al. 2018).

This type of coordination problem can not only be seen in many traditional foraging and hunting activities such as acquiring honey and hunting large prey but also in larger-scale international relations (Jervis 1978) and macroeconomics (Bryant 1994). Alvard and Nolin (2002) provide an illustrative example from the village of Lamalera, Indonesia, where fishermen regularly face the decision to either hunt sperm-whales in collaboration with others (viz. Rosseau’s Stag) or catch small fish individually with a hook-and-line or net (Rosseau’s Hare). They found that return rates from cooperative whale hunting were greater per capita than those from solitary fishing and that the distribution of the catch (shared payoff) was regulated by a mutually understood sharing-of-the-spoils normative framework. The general point is that this example of real-life collaboration is better framed as a SHG rather than a PDG as the chances of catching a whale individually are negligible and therefore not a tempting strategy.

While this kind of coordinated action may not be unique to humans (e.g., collaborative hunting in Lions *Panthera leo*, Stander 1992; Maynard-Smith & Szathmáry 1995; Chimpanzees *Pan troglodytes*, Boesch 2002; African wild dogs *Lycaon pictus* (Creel & Creel 1995) under some evolutionary accounts it played a pivotal role in evolution of humans’ status in the natural world as ultra co-operators. Essentially, humans became increasingly interdependent with one another such that each individual had a direct interest in the well-being of others or else they starved (Melis et al. 2006; Hamann et al. 2011; Warneken et al. 2011; Tomasello et al. 2012; Tomasello and Vaish 2013). The Stag Hunt therefore turns cooperation dilemmas into a problem of coordinating behaviour to achieve a mutual goal of mutual interest. One clear way in which humans are uniquely placed to reach sophisticated levels of coordination is through language, where intentions are made public (Schelling 1960; Lewis 1969; Duguid et al. 2014). Therefore, using an ABM, we first ask what is the highest energy expenditure environment that can sustain a population who differ in cooperative phenotypes (Hunt Stag or Hunt Hare) and then run the same simulation allowing agents to share their hunting intentions with a primitive language.

Previous modelling work has revealed complex evolutionary dynamics at work when the 2-person Stag–Hunt is generalised to a N-person game, specifically, scenarios of defector dominance, pure coordination, or coexistence may arise simultaneously if the population is assumed to be infinite, but when populations are finite and the population is the same size as the group, the evolutionary dynamics are profoundly affected, inverting the direction of natural selection (Pacheco et al. 2009). Building on this work Schnell and colleagues (under review) showed that the size of the Stag reward further affected the level of cooperation in a N-person game such that when a new cooperation threshold is accessible to a population, the level of cooperation increases to reach this threshold. However, when the next threshold is out of reach, cooperation decreases as individuals refrain from costly cooperation. Their findings have relevance for the leaps in economic development witnessed throughout human history, such as revolutions in the use of natural resources, the increasing manpower and infrastructure needed to land a whale, mine coal or refine oil which often required the cooperation of larger groups than those needed to burn wood or catch a stag.

More generally, evolutionary models of the SHGs have examined well-mixed, unstructured populations and uniform random pairing (Kandori et al. 1993; Young 1993; Ellison 1997; Sugden 2005); some degree of spatial structure or assortment (Ellison 1993; Skyrms 2003; Goyal and Vega-Redondo 2005; Staudigl and Weidenholzer 2014); or random encounters between agents either using assortment or not (Eshel and Cavalli-Sforza 1982; Bergstrom 2003; Allen and Nowak 2015). To our knowledge though, none of the studies above have included the ecological backdrop against which the evolutionary dynamics of SHGs play out. In this paper, we include this as a factor in our model, not only because it is important in the bigger story of the climatic and ecological drivers of hominin evolution, but by integrating an ecological landscape into the dynamics of game-theory payoffs, it can profoundly alter the outcome of what strategy is optimal for individuals.

In our ABM model, individual agents stay alive in the SHG when their foraging strategy results in energy income ≥ than their energy expenditure. If they stay alive long enough to reproduce, their offspring inherit the same hunting preference as their parents. The long-term, intergenerational survival of a phenotype is given by the aggregate success of individuals pursuing a particular fixed foraging behaviour (Hunt Stag or Hunt Hare). Individuals lose a constant amount of energy per unit of time from their reserves which can be increased or decreased to simulate harsher or more favourable environments respectively.

The output of the ABM will then inform the subsequent mathematical analysis where we focus on the dynamics of environmental harshness by dividing Stag hunters and Hare hunters into well-fed and poorly fed individuals, which combine in certain ways to produce different hunting success.

The long-term survival of fixed hunting phenotypes from the ABM and mathematical analysis raises the question of whether in real-life humans adopt a more flexible strategy, choosing to cooperate (or not) as a function of the environmental harshness and available energy reserves. We tested this hypothesis with an online version of the ABM where participants controlled the hunting decisions of their online avatars. The converging lines of evidence from the three approaches allow us to triangulate the importance of environmental variability in the SHG and its role in the evolution of human collaboration.

## METHODS

The methods associated with our three lines of enquiry – ABM, mathematical analysis, and the human stag hunt experiment – are each described in turn.

### The ABM model

#### Background

Our ABM implements several important generic features of an organism’s life, such as reproduction, mobility, resource accumulation, a cost-of-living, death, and a decoupling of birth and death events such that total population size can vary (Epstein 1998; Smaldino et al. 2013). By doing so we can examine the behaviour of repeated Stag–Hunt interactions when played out against a background of ecologically relevant constraints, such as the maximum species population size that is indefinitely sustainable, given the food, competition, and habitat available (Hui 2006). This allows complex population dynamics to emerge, such as the oscillatory cycles reminiscent of predator–prey dynamics when defectors exploit co-operators and then grow too concentrated to sustain their own inflated numbers (Nowak and May 1992; Smaldino et al. 2013).

The aim of the game is to stay alive long enough to reproduce. Individual agents stay alive when their foraging strategy results in energy income ≥ than their energy expenditure. The long-term, intergenerational survival of a population is given by the aggregate success of individuals pursuing a particular fixed foraging behaviour (hunt Stag or hunt Hare). The model can be played on the NetLogo agent-based platform and is freely available here https://ccl.northwestern.edu/netlogo/. For those wishing to replicate our results, the code for Model 1 and 2 is available in Supplementary Appendix 1.

#### Procedure

1. The simulation begins by populating a grid with *N* agents (*A*) who can move one cell at a time in any direction in search of food. Food appears in the form of *N* Stags *(S*) and *N* Hares (*H*).

2. An equal energy quota is deposited into each *A*’s account at the start of its life (*E*_s_), which could be thought of as initial birth weight or inherited fat reserve. From then on it loses a constant amount of energy per unit of time from that account as defined by the energy expenditure rate (*E*_r_). We chose to use “per time step” rather than “per cell moved” to reflect the fact that even when not moving, an organism expends energy to maintain metabolic life functions. This measure also implements the fact that in cold environments a relatively higher proportion of calories are spent maintaining these functions or in environments of scarce or poor quality resources food is harder to come by. In this way, varying energy expenditure rate can be used to simulate more favourable or harsher environments in which to live.

3. For each *A*, there is an upper maximum energy amount (*E*_max_) such that energy gains beyond that threshold will not add to an agent’s account total.

4. For each *A*, they are assigned a foraging strategy that remains the same for the duration of the simulation. An *A* can either only hunt Stags OR only hunt Hares. Both Hare Hunters and Stag Hunters exist on the grid at the same time.

5. All *A*’s randomly wander around the grid in search of food from the moment they are born until they die or the simulation is terminated, whichever happens first.

6. When an *A*_*i*_ meets another agent *A*_*j*_ in an adjacent cell of the grid, they choose to hunt Hare or Stag according to their foraging strategy as defined in (4). Steps 7–11 govern the outcome of such a meeting between all possible pairs

7. If both *A*_*i*_ and *A*_*j*_ are Stag Hunters they pair up occupying adjacent pairs of cells and wander the grid in search of food together. If they encounter a Stag they both receive the Stag payoff as defined in the Stag Hunt Payoff Matrix (Table 1). That payoff is added to their energy account.

**Table 1:** Classic Stag Hunt Payoff Matrix

		A_i_	
		Stag	Hare
A_j_	Stag	2,2	0,1
	Hare	1,0	1,1

8. In the no communication between agents scenario, if *A*_*i*_ is a Stag Hunter and meets *A*_*j*_ a Hare Hunter, then they pair such that *A*_*i*_ Stag Hunter follows *A*_*j*_ Hare Hunter around together but *A*_*i*_ Stag Hunter cannot receive a Stag Payoff if they encounter a Stag – by definition obtaining a Stag requires the cooperation of two agents on a Stag Strategy. If *A*_*j*_ Hare Hunter encounters a Hare, *A*_*j*_ gets to eat it. Thus *A*_*i*_ Stag Hunter pays a penalty by being locked into a partnership with no hope of obtaining food to offset *e*, for the time they are in that partnership. This is fundamentally why the optimal strategy is a matter of coordination and the Stag is the riskier, but payoff dominant strategy. In the scenario where communication is allowed between agents, a Stag Hunter can “query” the hunting intentions of their partner when they first meet and the potential partner is obliged to answer, thus there is a channel of communication between the two agents. After this initial query has been answered, a Stag Hunter will only commit to a hunt in the communicative scenario when intentions align (Stag–Stag) and will walk away from a potential hunt when they do not (Stag–Hare).

9. Vice versa if *A*_*i*_ is a Hare Hunter and meets *A*_*j*_ a Stag Hunter (see symmetrical payoff on the diagonal Table 1).

10. Stag–Hare partnerships end when either the Hare hunter catches a Hare, or either of the agents die of hunger, whichever occurs first.

11. If *A*_*i*_ is a Hare Hunter and meets *A*_*j*_ a Hare Hunter, then they immediately go their separate ways and continue looking for a Hare. After they have found one, they collect the Payoff then they wander the grid looking for more food.

12. Agents produce one offspring at a time and at a rate defined by *R* units of time. Offspring inherit their parents’ foraging strategy (see Table 2, Rationale for starting value for *R*).

**Table 2:** List of constants, variables, and the rationale for their starting values

Constants	Starting value	Rationale for starting value
Starting hunters energy (*E*_s_)	20	An equal energy quota is deposited into each *A*’s account at the start of its life (*E*_s_), which represents initial birth weight or inherited fat reserve. This value is high enough so that agents have an initial window of opportunity to catch food according to their hunting strategy and low enough such that there is a threat of starvation at some point in the simulation of the game if the hunting strategy is not successful.
Maximum hunters energy (*E*_max_)	20	For each *A*, there is an upper maximum energy amount (*E*_max_) such that energy gains beyond that threshold will not add to an agent’s account total. Even in species that deposit fat reserves as an insurance against harsh times, there are still upper limits to the amount of food they can metabolically store. There is a lower minimum energy amount such that agents below that will be eliminated from the grid: they have “starved” to death because their energy expenditure > energy gained through food.
Number of Stags to Hunt	100	The raw number of Stags is high enough to support a Stag Hunting Strategy yet low enough to ensure that the Energy Expenditure Rate (*E*_r_) is a determining factor in the survival chances of Stag Hunters. The number of Stags to Hares was matched to isolate the influence of Energy Expenditure Rate and Stag/Hare Payoff Ratios.
Number of Hares to Hunt	100	The raw number of Hares is high enough to support a Hare Hunting Strategy yet low enough to ensure that the Energy Expenditure Rate (*E*_r_) is still a determining factor in the survival chances of Hare Hunters. The number of Hares to Stags was matched to isolate the influence of Energy Expenditure Rate and Stag/Hare Payoff Ratios.
Stag and Hare Introduction Rate (*F*)	1.0	*F* is high enough such the replacement rate of either Stags or Hares in the environment will support their respective hunting strategies and low enough to ensure that the Energy Expenditure Rate (*E*_r_) is still a determining factor in the survival chances of either Stag or Hare Hunters.
Number of Stag Hunters	100	The number of Stag Hunters is large enough such that, in comparison with the number of Stags, this strategy is sustainable by providing enough potential partners to collaborate with. It is matched with number of Hare hunters (see below) to isolate the influence of Energy Expenditure Rate and Stag/Hare Payoff Ratios.
Number of Hare Hunters	100	This value is matched with number of Stag Hunters (see above)to isolate the influence of Energy Expenditure Rate and Stag/Hare Payoff Ratios.
Reproduction Rate (*R*)	1000	For all the simulations we investigate we allow the reproduction rate to be greater than the starting energy divided by the energy expenditure. This ensures that the relative payoffs become a relevant factor for agent survival. Without such a constraint the population is infinitely sustainable with no energy input, which is obviously unrealistic. This is because their initial energy deposit would be enough to sustain life until their offspring received the initial energy deposit and so on without any need to obtain food in the meantime.
Variables		
Stag/Hare Payoff Ratio	1:1, 2:1, 3:1, 4:1, 5:1	A variable Stag/Hare payoff ratio was chosen (a) to reflect the fact that an optimal strategy in Game Theory often depends on the exact ratio of the payoff matrix (b) starting the ratio at 1:1 provides a baseline to examine any inherent Stag- or Hare-hunting advantage in foraging strategy when the payoffs are kept constant and (c) as the results will show, it gives us a range of survival probabilities from 0 to 1 that will explore the limits of cooperation for the chosen environmental stress range. Exactly how much bigger the payoff of a Stag is relative to the Hare is a variable that affects how tolerable harsh conditions are; therefore we varied this from 1:1 no advantage to 5:1 a large advantage (cf. “The Size of the Stag Determines the Level of Cooperation” Schnell and colleagues (under review))
Energy Expenditure Rate (*E*_r_)	0.02, 0.03, 0.04, 0.05, 0.06, 0.07, 0.08, 0.09, 0.1, 0.11, 0.12	A variable energy expenditure rate is chosen (a) to begin at a lower limit that would satisfy the Reproduction Rate constraint and (b) to extend to an upper limit where, no matter what the strategy or payoff, all hunters would eventually die. Thus, we can be sure we had explored the full range of outcomes relevant to our main research questions.

**Figure 1:**
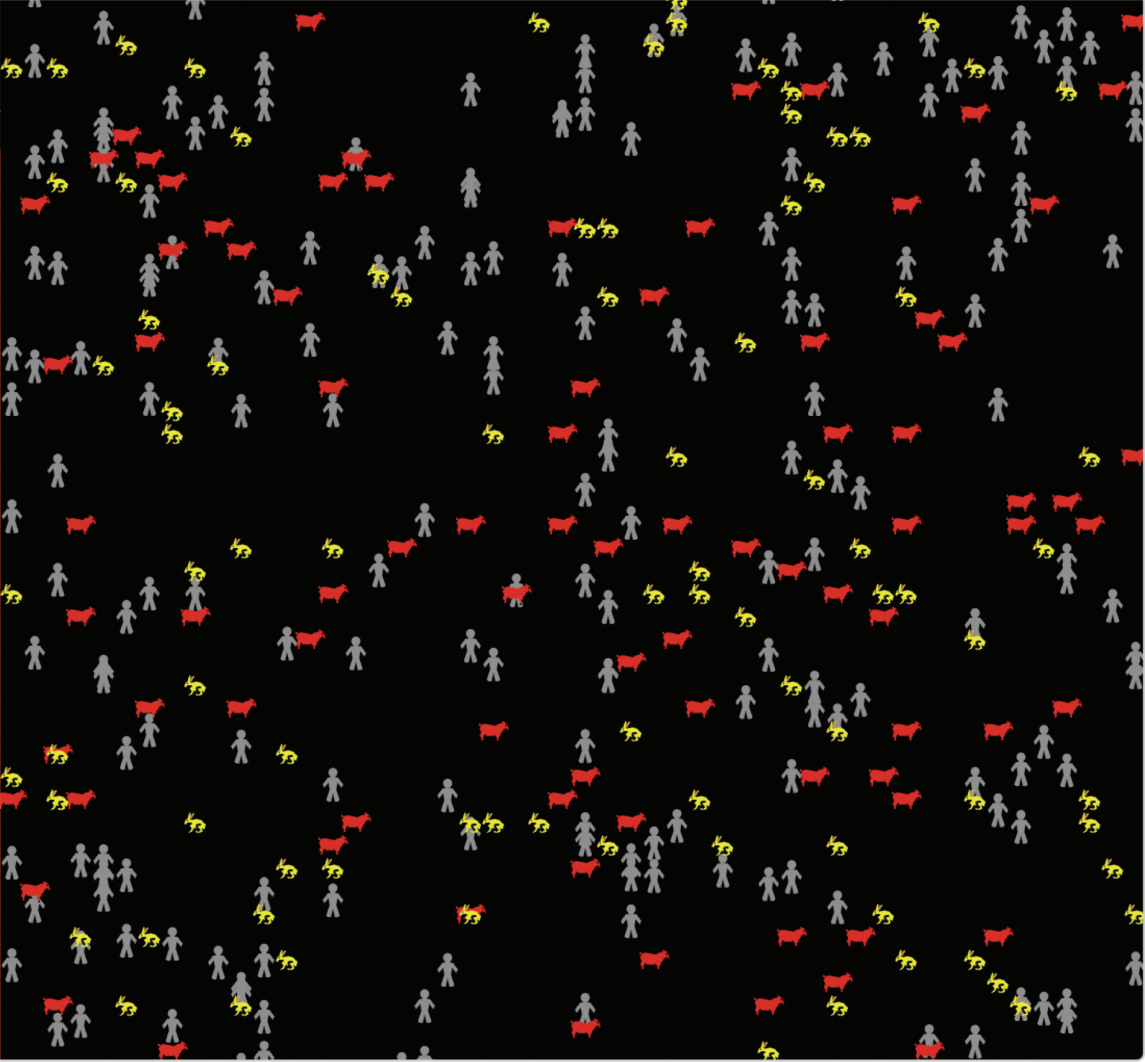
Grid of Stags (red animals) and Hares (yellow) and Agents (grey characters). The game can be played on this freely available software https://ccl.northwestern.edu/netlogo/ with the code available in Supplementary Appendix 1.

13. Both Stags and Hares walk across the grid from top to bottom at a given rate *F* (number of Stags and Hares introduced to the grid per time unit) and if they are captured by a hunter before they exit the bottom of the grid they are not replaced. In other words, the food available to hunt has a finite, temporal availability, until it is replaced at rate *R*. Note that because the resources in our model do not replicate themselves, the results can be generalised to coordination games with similar payoff structures but that do not involve replicating prey, such as procuring honey (viz. Stag) versus berries (viz. Hare).

14. In all cases if an *A* fails to find food before reaching their lethal lower limit (energy account = 0) they are removed from the grid and not replaced.

15. If an *A*_*i*_ is unlucky enough to be paired with another *A*_*j*_ who dies in the process of looking for food together, then *A*_*i*_ decouples and begins looking for another partner to play, but they having lost the energy expended during the partnership (Figure 1).

Constants, variables, and rationale for starting values.

Table 2 details the constants and the variables we used in our ABM. For these values, we record the probability (0–1) of agents with a given foraging strategy (hunt Hare, hunt Stag) being alive after 50 000 units of time. The probability of survival was averaged over a 100 simulation runs for each of the payoff and environmental harshness variables. The general rationale for choosing the starting values was to select those constants that stabilised the dynamics of the game for longer enough to measure the effect of the variables we were interested in.

### Mathematical analysis

To draw formal generalisations from the results of the ABM, we denoted the population density of unattached stag-hunters by S, that of unattached hare-hunters by H, that of paired stag-hunters by S_2_, and that of stag-hunters paired with hare-hunters by B. We denoted by F_s_ and F_h_ the quantities of stags and hares.

We assumed that free hunters interact at rate, k, to form pairs. We assumed that heterotypic pairs (Stag–Hare) resolve when the hare-hunter catches a hare or when either of the pair dies. We assumed that pairs of stag-hunters resolve when a stag is caught or one of the pair dies. The time taken for pairs to resolve depends on the energy levels that the individuals have at the start of their union and on the harshness of the environment. We assumed that pairs only resolve if their energy levels drop too low or they catch prey.

All individuals reproduce at rate 1/R. We assumed that if individuals in a pair reproduce, their offspring are no longer paired. Stags and hares are provided at rate F and leave the domain at a rate of l_3_ times their levels. We assumed the population is well-mixed, so each hunter has access to the current level of food and pairs form at uniform rates throughout the domain. We divided S and H into well-fed and poorly-fed individuals, which combine as follows:


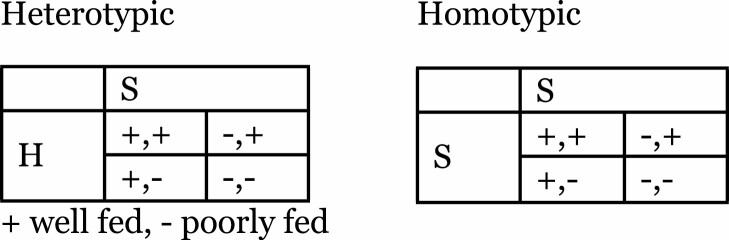


We assume poorly fed stag-hunters and hare-hunters and poorly fed individuals in pairs have constant death rates (l_1_ and l_2_ respectively). The equations for the different types of hunters, food, and pairs are given in the Mathematical Details.

Hare-hunters transition from the well-fed to poorly-fed at the same rate at which poorly-fed individuals die. Stag-hunters transition from the well-fed group into the poorly-fed group at a lower rate al_1_. α < 1 reflects the slower passage of stag-hunters to the poorly-fed group, because their food is of higher value.

We assumed when pairs reproduce, their offspring are initially well-fed. When a pair of stag-hunters catches a stag, they become well-fed individuals. When a hare-hunter paired with a stag-hunter catches a hare, a well-fed hare-hunter results. The paired stag-hunter retains its well- or poorly-fed status. Similarly, when an individual in a pair dies, its partner retains its well- or poorly-fed status.

The rate of degradation of the environment is important in comparison to the rate at which food is caught. We therefore vary k_e_. k_e_ small corresponds to a harsh environment, where energy is lost fast compared with the food catching rate and k_e_ large to a favourable environment, where energy is lost comparatively slowly.

### The human stag hunt experiment

#### Participants

132 participants (mean age, 32.80, SD = 7.88; 86.86% female) enrolled in undergraduate psychology and childhood studies courses at a UK Higher Education institute voluntarily took part in the study with no compensation offered and were recruited via an open invitation, advertised on their module homepage.

#### Ethics

The project received favourable approval from lead researcher’s institute’s The Open University Human Research Ethics Committee – reference number: HREC/3553/Ibbotson, approved 29/05/2020. All participants were informed as to the purpose of the study, freely consented, were assured no individual data could be identified from any publication resulting from the study, free to withdraw from the experiment at any time, and were offered to be informed of the results after the experiment had closed to all participants.

#### Procedure

If participants consented, they were given a link to play a cooperation game online, designed using a modification of the NetLogo program used in the ABM to allow interactive harvesting of data and hosted on the university data server. Once on the landing page of the experiment they received the following instructions:

“You are going to play a series of online games where your task is to try to stay alive. To play the game, enter your gender and age, press ‘setup new game’ and press ‘play’. You automatically walk around the square below looking for food – you are the little orange person and other hunters are in grey. You lose energy by walking around looking for food, you gain energy when you find food. You stay alive by making sure the energy you gain from your food is more than the energy you use to find it. Once the game starts, you decide whether to hunt a Hare or a Stag. You can switch between hunting Hares and Stags in the game to maximise your chances of staying alive. You can catch the Hare on your own. However, the Stag is bigger and to catch it you need to find another hunter who is also wants to hunt a Stag. If you choose to hunt a Hare you have a 100% chance that you will get 100 calories from the Hare if you catch it. If you choose to hunt a Stag, you have a 50% chance that you will get 200 calories from a Stag. On the left-hand side of the screen, you will see your energy reserves in a bar. The bar goes down when you search for food and when the bar reaches the bottom, you have starved to death. The aim of the game is to be alive by the end of the game”.

**Figure 2:**
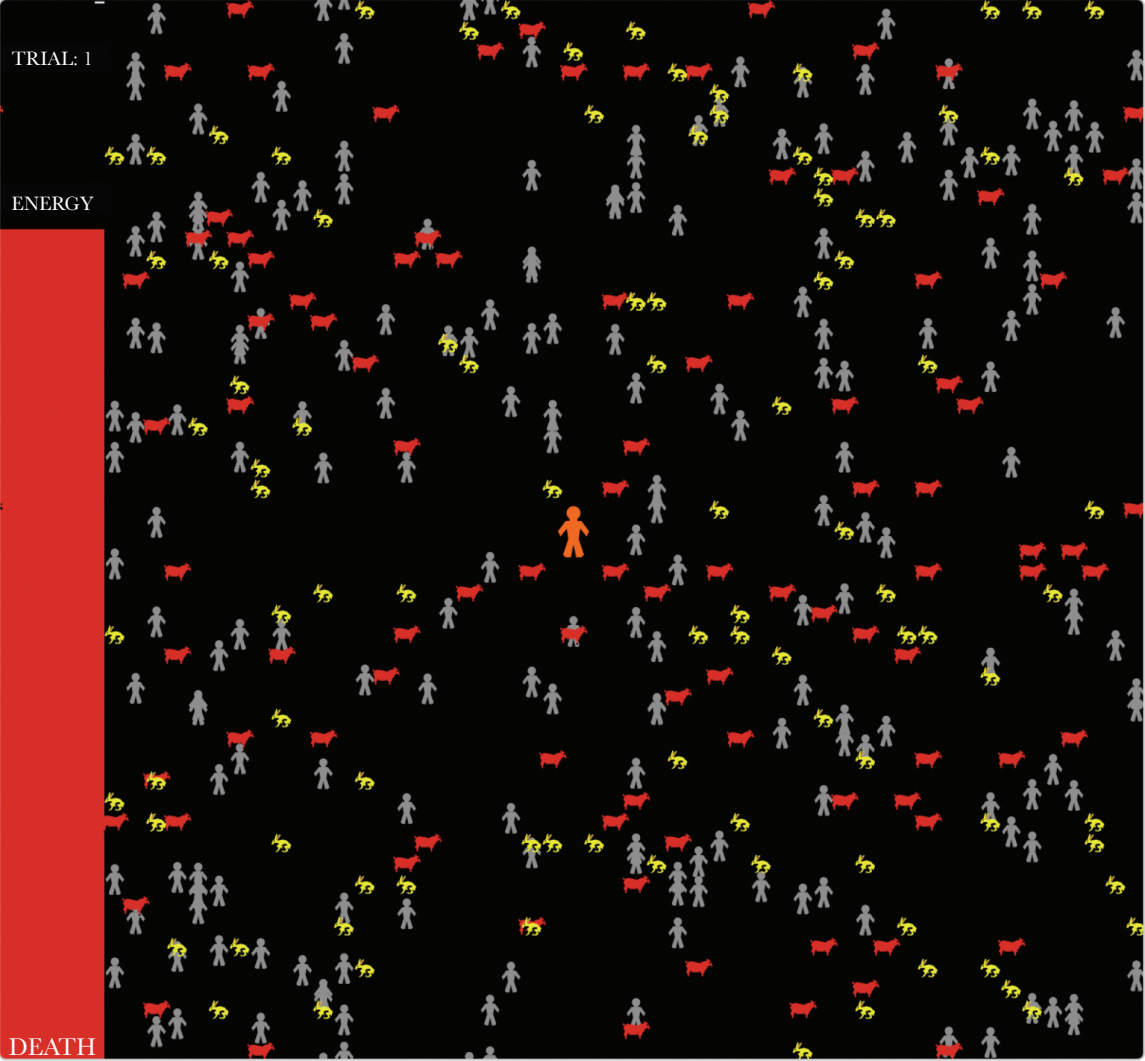
A screenshot of Human Stag Hunt experiment, with the online avatar (orange character in the middle of the screen), other hunters (grey characters) Stags (red animals), and Hares (yellow). Also available online were buttons to select their hunting choice (Stag or Hare).

Participants monitored the progress of their hunting choices as their onscreen avatar wandered the hunting grid looking for food (screenshot in Figure 2). The energy bar decreased as a proportion of the background environmental harshness assigned to that trial (see below) and increased as a proportion of the food captured: 2 units for a Stag and 1 for a Hare.

Each participant took part in 6 trials in which the energy decay rate was either 0.02, 0.025, 0.03, 0.035, 0.04, the order of which was randomised for each participant. Once participants had completed their set number of trials, they submitted their data to a secure server which was later collected by the experimenters for statistical analysis.

## RESULTS

The results associated with our three lines of enquiry – ABM, Mathematical Analysis, and The Human Stag Hunt Experiment – are each described in turn.

### Stag hunt ABM results

**Figure 3:**
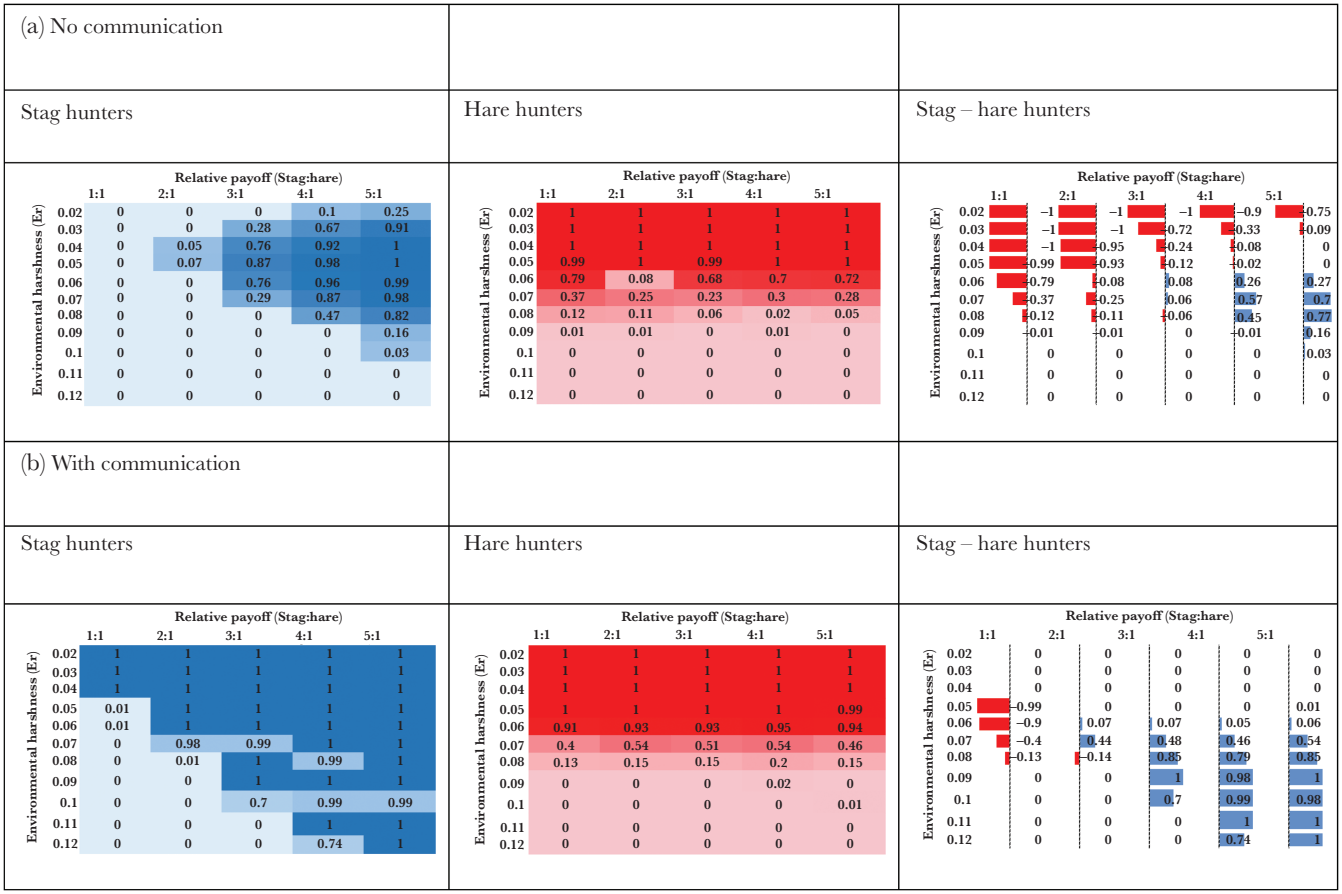
**(A)** No communication allowed between agents. Numbers indicate the survival probability (0–1) of a population being alive after 50 000 units of model time as a function of Environmental Harshness and Relative Payoff (Stag:Hare). The first column summarises results for Stag Hunters (blue), the second column summarises results for Hare hunters (red), and the third column are the results of subtracting Stag hunting results from the Hare hunting data. In this third column, red bars (negative numbers) represent the size of the survival advantage for Hare hunting, Blue bars (positive numbers) represent the size of the advantage for Stag hunting and no bars (0) represents no overall advantage for either Hare hunters or Stag hunters. **(B)** The same procedure as in (A) but with communication allowed between agents.

The output of the ABM gave probability (0–1) of agents with a given foraging strategy (hunt Hare, hunt Stag) being alive after 50 000 units of time. The probability of survival was averaged over a 100 simulation runs for each of the payoff and environmental harshness variables and are reported in Figure 3 (for a 3-dimensional representation of the same data see Supplementary Appendix 2).

### Mathematical analysis

**Figure 4:**
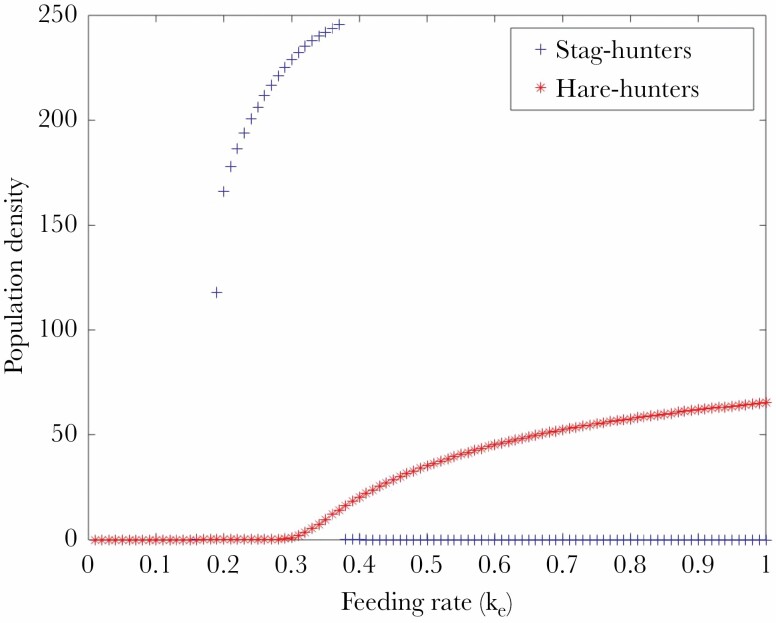
Steady state levels of stag- and hare-hunters as the harshness of the environment is modified by varying the food-catching rate k_e_. Small values of k_e_ correspond to a harsh environment.

The mathematical analysis shows how the steady state levels of hare-hunters and stag-hunters change with k_e_. We see that a harsh environment favours stag-hunters and a more favourable environment favours hare-hunters (Figure 4). We note that the nutritional value of a stag is much greater than that of a hare. We model this by assuming that the rate at which stag-hunters transition from the well-fed to the poorly fed category is reduced. This is quantified by the parameter α. The steady state level of stag-hunters, in the absence of hare-hunters is very sensitive to this parameter. When α is sufficiently large, hare-hunters always win. When α is smaller, stag-hunters can win at intermediate feeding rates, and their steady state levels are very high, because they transition slowly to the poorly-fed category.

### The human stag hunt results

We analysed the results of the online cooperation game using binary logistic regression as our outcome variable had two possible states: switch from Stag hunting to Hare hunting or switch from Hare hunting to Stag hunting. There was no issue of multicollinearity as we only had one independent variable: current energy (the amount of red bar players had left when they made their hunting switch) and because of this the sample size adequately powered the analysis where the general guidelines suggest a minimum of 10 cases with the least frequent outcome for each independent variable in the model. Our data represented 3098 hunting decisions. To simplify the analysis, we divided the analysis into a “good times” group, equivalent to the ABM model of environmental harshness where *E*_r_ 0.02–0.025, and the energy depletion was relatively slight, and a “hard times” group, where *E*_r_ 0.03–0.4, and the energy depletion occurred at a quicker rate and thus represented “harder times” or a more challenging ecological landscape against which to hunt and stay alive. These boundaries were chosen as the result of piloting trial and error with the target stimuli and reflected the need for the environmental background to be sustainable enough to allow human participants in the game enough time to decide on a hunting strategy (i.e., not overly harsh) but also challenging enough so that environmental background might impinge on the decision they made (i.e., not overly favourable).

A binomial regression analysis resulted in significant effects of current energy in predicting whether humans would cooperate or not: In “Good times” energy levels predicted strategy switch χ2 (1) = 49.02, *P* < 0.001, such that every unit increase in energy increased the logs odd for a Hare to Stag switch by −0.06, *P* < 0.001, 95% CIs [0.92, 0.95], pseudo R^2^ measures: Cox & Snell = 0.027, Nagelkerke = 0.036. In “Hard Times” energy levels predicted strategy switch χ2 (1) = 290.13, *P* < 0.001, such that every unit increase in energy increased the logs odd for a Hare to Stag switch by −0.19, *P* < 0.001, 95% CIs [0.80, 0.084], pseudo R^2^ measures: Cox & Snell = 0.2, Nagelkerke = 0.268. For an analysis that addresses pseudoreplication concerns with data, please see Supplementary Appendix 3.

**Figure 5:**
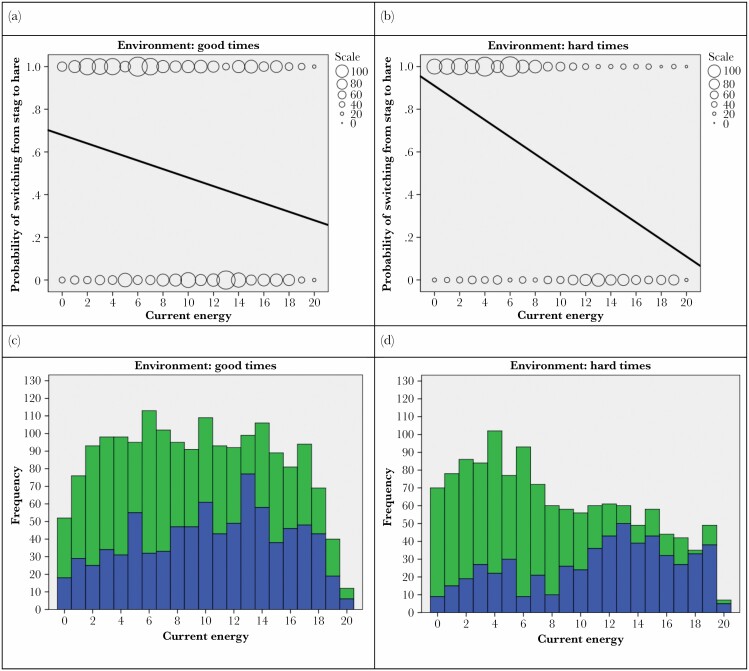
Binomial regression of predicted probabilities of switching from Hare to Stag for Good Times (*y* = 0.68−0.02*x) (**A**) and Hard Times (*y* = 0.91−0.04*x) (**B**) (circles indicate the frequency of responses in each bin). The histograms display the raw frequency data on which the predicted probabilities are calculated for Good Times (**C**) and Hard Times (**D**) where green bars represent a switch from Hare to Stag and blue bars represent a switch from Stag to Hare (note bars are not overlapping but cumulative).

To test whether there was an overall significant effect of environmental harshness, this was entered separately into a binomial regression analysis resulted in significant effects of environment in predicting when humans would cooperate or not: such that environment predicted strategy switch χ2 (1) = 4.403, *P* = 0.03, such that every unit increase in energy increased the logs odd for a switch by −0.154, *P* < 0.001, 95% CIs [0.743, 0.99], pseudo R^2^ measures: Cox & Snell = 0.01, Nagelkerke = 0.002 (Figure 5).

## DISCUSSION

The results and their implications for each of our three lines of enquiry – ABM, Mathematical Analysis, and The Human Stag Hunt Experiment – are discussed in turn below, before finally drawing some general conclusions.

### ABM

Results from the ABM model show that what is optimal for an individual's foraging behaviour in an SHG depends not only on the individual’s own actions and the behaviour of other foragers, but also the environmental backdrop against which these phenotypes compete. In the No Communication scenario, Hare hunting is dominant at low levels of environmental harshness (*E*_r_ 0.02–0.06) but as conditions worsen (*E*_r_ 07–0.09) and the payoff of a Stag increases relative to Hare, Stag Hunting phenotypes are ever more likely to survive than Hare Hunters and are the only viable strategy to survive where *E*_r_ = 0.1 (Figure 3, 3^rd^ Column, Stag – Hare Hunters). In the Communication scenario, there is no overall advantage for Stag Hunters or Hare Hunters at low levels of environmental harshness (*E*_r_ 0.02–0.06) but that situation changes to being dominated by Stag Hunter survival when condition worsens (*E*_r_ > 0.06) including being able to survive in the harshest environment tested (*E*_r_ 0.12). This pattern is amplified at higher levels of Stag payoffs (>3:1), which accords with more general findings that the payoff ratios influence the selection of the risk or the payoff-dominant equilibria in the SHG (Battalio et al. 2001; Schmidt et al. 2003; Rydval and Ortmann 2005; Devetag and Ortmann 2007).

**Figure 6:**
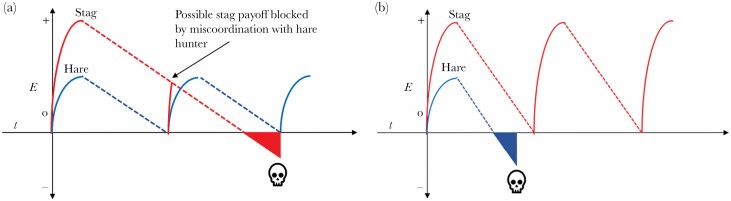
In favourable environments **(A)** Stag Hunters pay the penalty for miscoordination when they are coupled with a Hare hunter because the Stag hunter has no chance of offsetting his energy expenditure with a Stag capture while they remain a pair. In harsh environments **(B)**, the payoff returns of a Hare are not sustainable. The more Hare Hunters that die off in the harsh times, the less opportunity there is for miscoordination and so further improves the Stag hunters’ chances of survival.

Following a similar line of reasoning to the cost-benefits analysis of optimal foraging theory (Krebs 1977), we propose the mechanisms driving these results are as follows. Cooperating by hunting and sharing-the-spoils of a large prey is the only way to survive harsh environments because of the steep decline in energy levels needs to be offset by a large payoff to avoid starvation (i.e., “dying to cooperate”). However, when the environment is more favourable, because energy expenditure is lower, Hare Hunting becomes a viable strategy which in turn creates a lot of “nuisance” potential pairs that will waste the time and energy of Stag hunters once committed to a fruitless collaboration (Figure 6). This provides insight into an aspect of cooperation previous models have failed to account (Andras et al. 2007): if cooperation dominates in harsh environments why does it not also pay during favourable ones.

When agents could communicate their hunting intentions, Stag Hunting was more successful than Hare Hunting in almost all environmental scenarios and payoff ratios examined. Communication enabled Stag Hunters to walk away from a potential collaboration if their strategy did not align with those of their partner and avoid the miscoordination penalty. This provides a strong selection pressure in favour of sharing communicative intentions and in line with evolutionary accounts of human cooperation that stress mutual interdependence (Melis et al. 2006; Hamann et al. 2011; Warneken et al. 2011; Tomasello et al. 2012).

To return to the Indonesian sperm-whale hunters described in Alvard and Nolin (2002), they noted that fisherman engaged in extensive pre-hunt communication, making known their hunting intention and public commitment to the normative framework that would regulate any subsequent sharing-of-the-spoils. Here we have demonstrated that this communication allows cooperative phenotypes to withstand harsher environments. Note that for the conditions in which Hare Hunting is still the preferred strategy, even under communicative conditions, the explanation is also because of the price of miscoordination. Stag Hunters still need to probe others as to their intentions and this comes with a non-negligible cost of time and energy. Hare hunters can ignore the enquiry to cooperate and go about their business hunting Hares. Thus the advantage of Stag Hunting could be further increased in this model if there were some source of cheap, public, and honest perceptual information about potential collaborators, *before* they have to ask, such as that which exists in the green beard effect (Hamilton 1964; Queller et al. 2003).

It is interesting to consider whether the results would extend to PDGs. As we and others have argued, SHGs represent a better model for the evolution of human cooperation because it is a tighter fit to the real-life ecological and environmental challenges faced by our ancestors and many people today. The unsuitability of this model to ecological contexts becomes apparent if we were to naively transplant the classic Prisoner’s Dilemma payoff matrix into the narrative of the Stag Hunt. In this situation, the benefits of hunting a Hare when the other hunter chooses to hunt Stag, would be greater than the rewards for hunting a Stag together or hunting a Hare alone. Clearly, the analogy breaks down at this point, but we can ask, what role environmental harshness might play in SHGs. In the absence of other factors that have been shown to increase the chances of cooperation, such as communication, punishment, reward, or reputation, the dominant strategy is to defect in PDGs: the only outcome from which each player could only do worse by unilaterally changing strategy. While a full modelling of this scenario is beyond the scope of this paper, it seems reasonable to project from these results and the mechanisms laid out in Figure 6 that mutual defectors would be more harshly affected by adverse environmental conditions than would mutual cooperators as their payoff is greater (and thus why the mutual defection is the “tragic” dominant strategy). Simply put, if mutual cooperators are starting from a higher metabolic position, then they can sustain the cost-of-living for longer. However, the temptation to defect is still present in a way that it is not for the Stag Hunt (see above) so further investigations are needed to uncover exactly how Prisoner’s Dilemma would play out against varying degrees of environmental harshness.

### Mathematical analysis

The mathematical analysis synthesises what we understand to be the relevant factors from the output of the ABM. It shows how the steady state levels of hare-hunters and stag-hunters change with k_e_. We see that a harsh environment favours stag-hunters and a more favourable environment favours hare-hunters (Figure 4). The steady state stag-hunter level is a very sensitive to the parameter α, which is the ratio of the rate at which well-fed individuals become poorly-fed to the death rate of poorly-fed individuals. When this parameter is sufficiently small (corresponding to high nutritional value of a stag), the stag-hunters can sustain very high levels at lower feeding rates. At higher feeding rates, the hare-hunters become viable and pair up with stag-hunters, thus preventing the latter from forming homotypic pairs and catching food.

In our model, individuals do not switch/choose their behaviour, but rather survive or die depending on their strategy and the composition of the population. At very high pairing rates, most individuals will exist in pairs and so the death rates of stag-hunters will be determined by the probability that they are in homotypic (stag-catching) versus heterotypic pairs. In this case, our ecological model becomes more comparable to the two-player game analyses, with stag-hunting becoming the better strategy only when stag-hunters exceed a critical proportion.

### The human stag hunt experiment

The long-term survival of fixed hunting phenotypes from the ABM and formal analysis raised the question of whether, in real-life, humans adopt a more flexible strategy, choosing to cooperate (or not) dependent on the environmental harshness and available energy reserves. We tested this hypothesis with an online version of the ABM where participants controlled the hunting decisions of their online avatars. They monitored their energy reserves on screen which increased after a successful hunt and decreased as time elapsed. Hunting a Hare gave them a 100% chance of 100 calories whereas hunting Stag would give a 50% chance of 200 calories (replicating the classic 2:1 Stag Hunt ratio from the ABM). We systematically varied the environmental harshness for each participant and, as in the ABM, human players of the game lost energy by walking around looking for food and stayed alive by making sure the energy they gained from their food was greater than the energy they used to find it.

We found that the less energy people had in their reserves, the more likely they were to choose the collaborative option of Stag Hunting. Moreover, the harsher the environment the more amplified this effect became because it essentially heightened the threat of starvation and brought forward the decision to collaborate (“good times”/“bad times” comparison). Presumably, this was because participants were monitoring the state of their energy reserves in comparison to their current strategy and made decisions about optimal hunting strategies in the moment. Interestingly, recent evidence from behavioural economics also suggests there are fundamentally different motivations underlying decision making in one-shot PDGs versus SHGs, with PD cooperation governed by a strong moral component to “do the right thing” by your collaborator (e.g., Capraro and Rand 2018) whereas cooperation in SHGs is driven more by distributional, social preferences for efficiency (Capraro et al. 2020).

In the face of cooperative uncertainty – participants could not be certain all attempts at collaboration would be reciprocated – they favoured the high-risk high-reward Stag option when environments were harsher and starvation was imminent. In this on-the-fly cost-benefits analysis, our human participants behaved like other species placed in similar ecological positions. For example, when choosing between food sources with varying quality, foraging animals are predicted to select those that maximize their net energy intake by balancing the potential calorific payoff of the food reward with the risks or costs associated with obtaining it (Stephens 1986). It has been shown that the probability of engaging in risky foraging behaviors can also be influenced by an individual’s hunger level or energetic state (Hileman et al. 1994; Lima 1998; Gillette et al. 2000; Brown and Kotler 2004; Verdolin 2006).

As one might expect, organisms appear to accept greater risks when starvation is imminent or highly likely (Gotceitas and Godin 1991; Pettersson and Brönmark 1993; Godin and Crossman 1994). Riskier foraging strategies as a result of decreased energy levels have been observed in sparrows (*Junco hyemalis*) (Lima 1988), salmon (*Oncorhynchus kisutch*) (Dill & Fraser 1984), and slime mold (*Physarum polycephalum*) (Latty & Beekman 2010). It is known that species can mitigate their extinction risks in uncertain environments by diversifying individual phenotypes, in a process of so-called “bet hedging” (Nowak 2006). Thus different animal personalities, behavioural strategies, and syndromes (a proclivity to cooperate or not for example) can be thought of spreading the risk of uncertain environmental conditions across different phenotypes adapted to different environments (Chapple et al. 2012; Sih et al. 2012; Wolf and Weissing 2012; Carere and Gherardi 2013). The fact different kingdoms of life, and different levels of biological organisation, show similar responses to environmental stress suggests some common principles at work. However, where humans are of course different is that we have evolved to use language, which as the ABM results show vastly reduces the risk in Stag Hunt scenarios and in real life regulates the social contract in SHGs.

## CONCLUSIONS

The modelling and mathematical results show that only cooperative foraging phenotypes survive the harshest of environments but pay a penalty for miscoordination in favourable environments. Because of the presence of nuisance pairs (from the perspective of the Stag Hunters), we are able to explain why cooperation does not always pay during favourable environments, even if it does so in harsher environments. When agents are allowed to coordinate their hunting intentions by communicating, cooperative phenotypes outcompete those who pursue individual strategies in almost all environmental and payoff scenarios examined. This is important as compared with the rest of the natural world, not only are humans an ultra-cooperative species, they are an ultra-communicative one too. Under some evolutionary accounts, these two aspects are related via an adaptation for mutual knowledge where language can be thought of as “intention made public” (Schelling 1960; Duffy and Feltovich 2002; Smith 2010). As Alvard and Nolan note “the adaptive value of being able to communicate honest cooperative intent with such a statement as ‘I will hunt whales tomorrow with you if you hunt whales tomorrow with me’ is hard to overestimate.” (2002, p. 549) and along with them we also agree that because pregame communication is so crucial to the solution of coordination games such SHGs, this may have been one selective pressure favouring the evolution of language. The idea that language evolved in order to facilitate the planning involved in hunting is not new (e.g., Washburn and Lancaster 1968; Montagu 1976), but framing the issue in terms of environmental harshness here has helped to sharpen the role of communication further.

Our human data shows flexible decision-making in the face of cooperative uncertainty, with people favouring high-risk high-rewards when environments are harsher and starvation is imminent. We found that not only are humans sensitive to such contextual factors, they have the potential to make decisions about whether to cooperate or not that are optimal to our survival. While the cognitive biases that underpin coordination may have been selected for in life-or-death hunting situations of our past, modern-day humans still use the cognitive intuitions to resolve coordination problems whenever they have a structure and logic comparable to the Stag Hunt, such as those in larger-scale international relations (Jervis 1978) and macroeconomics (Bryant 1994). Converging lines of evidence from the three approaches we have taken here indicate a significant role for environmental variability in human cooperative dynamics and the species-unique cognition designed to support it.

## MATHEMATICAL DETAILS

We obtain the following equations for the time evolution of the variables:


$$\begin{array}{l} \frac{dF_{s}}{dt}=F-2k_{e}\left(S_{2}^{++}+S_{2}^{+-}+S_{2}^{--}\right)F_{s}-l_{3}F_{s} \end{array}$$

$$\begin{array}{l} \frac{dF_{h}}{dt}=F-k_{e}\left(B^{++}+B^{+-}+B^{-+}+B^{--}+H_{+}+H_{-}\right)F_{h}-l_{3}F_{h}\;\;\; \end{array}$$

$$\begin{array}{l} \begin{array}{ll} & \frac{dS_{+}}{dt}=\frac{6S_{2}^{++}+4S_{2}^{+-}+2S_{2}^{--}+3B^{++}+3B^{+-}+B^{-+}+B^{--}+S_{+}+S_{-}}{R}\\ & \;+4k_{e}\left(S_{2}^{++}+S_{2}^{+-}+S_{2}^{--}\right)F_{s}+k_{e}\left(B^{++}+B^{+-}\right)F_{h}-\alpha l_{1}S_{+}\\ & \;-kS_{+}\left(S_{+}+S_{-}\right)-kS_{+}\left(H_{+}+H_{-}\right)+l_{1}S_{2}^{+-}+l_{2}B^{+-} \end{array} \end{array}$$

$$\begin{array}{l} \begin{array}{ll} & \frac{dS_{-}}{dt}=\frac{2S_{2}^{+-}+4S_{2}^{--}+2B^{-+}+2B^{--}}{R}+k_{e}\left(B^{-+}+B^{--}\right)F_{h}+\alpha l_{1}S_{+}-l_{1}S_{-}-kS_{-}\left(S_{+}+S_{-}\right)\\ & \;-kS_{-}\left(H_{+}+H_{-}\right)+2l_{1}S_{2}^{--}+l_{2}B^{--} \end{array} \end{array}$$

$$\begin{array}{l} \begin{array}{ll} & \frac{dH_{+}}{dt}=\frac{3B^{++}+3B^{-+}+B^{+-}+B^{--}+H_{+}+H_{-}}{R}+k_{e}\left(B^{++}+B^{+-}+B^{-+}+B^{--}+H_{-}\right)F_{h}\\ & \;-l_{2}H_{+}-kH_{+}\left(S_{+}+S_{-}\right)+l_{1}B^{-+} \end{array} \end{array}$$

$$\begin{array}{l} \frac{dH_{-}}{dt}=\frac{2B^{+-}+2B^{--}}{R}+l_{2}H_{+}-k_{e}H_{-}F_{h}-l_{2}H_{-}-kH_{-}\left(S_{+}+S_{-}\right)+l_{1}B^{--} \end{array}$$

$$\begin{array}{l} \frac{dS_{2}^{++}}{dt}=\frac{kS_{+}^{2}}{2}-\frac{2S_{2}^{++}}{R}-2k_{e}S_{2}^{++}F_{s}-2\alpha l_{1}S_{2}^{++} \end{array}$$

$$\begin{array}{l} \frac{dS_{2}^{+-}}{dt}=kS_{+}S_{-}-\frac{2S_{2}^{+-}}{R}-2k_{e}S_{2}^{+-}F_{s}-\left(1+\alpha \right)l_{1}S_{2}^{+-}+2\alpha l_{1}S_{2}^{++} \end{array}$$

$$\begin{array}{l} \frac{dS_{2}^{--}}{dt}=\frac{kS_{-}^{2}}{2}-\frac{2S_{2}^{--}}{R}-2k_{e}S_{2}^{--}F_{s}-2l_{1}S_{2}^{--}+\alpha l_{1}S_{2}^{+-} \end{array}$$

$$\begin{array}{l} \frac{dB^{++}}{dt}=kS_{+}H_{+}-\frac{2B^{++}}{R}-k_{e}B^{++}F_{h}-\left(l_{2}+\alpha l_{1}\right)B^{++} \end{array}$$

$$\begin{array}{l} \frac{dB^{+-}}{dt}=kS_{+}H_{-}-\frac{2B^{+-}}{R}-k_{e}B^{+-}F_{h}-\left(l_{2}+\alpha l_{1}\right)B^{+-}+l_{2}B^{++} \end{array}$$

$$\begin{array}{l} \frac{dB^{-+}}{dt}=kS_{-}H_{+}-\frac{2B^{-+}}{R}-k_{e}B^{-+}F_{h}-\left(l_{2}+l_{1}\right)B^{-+}+\alpha l_{1}B^{++} \end{array}$$

$$\begin{array}{l} \frac{dB^{--}}{dt}=kS_{-}H_{-}-\frac{2B^{--}}{R}-k_{e}B^{--}F_{h}-\left(l_{2}+l_{1}\right)B^{--}+\alpha l_{1}B^{+-}+l_{2}B^{-+} \end{array}$$

We perform numerical simulations of the model, using the Matlab ordinary differential equation solver ODE45 and the following parameter values *F* = 10.0, *R* = 2.0, *k* = 0.6, *α* = 0.75, *l*_1_ = 1.2, *l*_2_ = 1.05, and *l*_3_ = 30.0. We use initial conditions *F*_*s*_(0) = *F*_*h*_(0) = 10, *S*_+_(0) = *H*_+_(0) = 10, *S*_−_(0) = *H*_−_(0) = *B*(0) = *S*_2_(0) = 0.

We note that if we make the environment sufficiently favourable, stag-hunters re-emerge.

The total levels of hare-hunters do not depend on the level of stag-hunters, so it is possible to understand the dynamics of hare-hunters in isolation. We find that hare-hunters converge to a steady state level given by


$$\begin{array}{l} H_{TOT}=\left[-\frac{l_{3}}{k_{e}}+\frac{F}{l_{2}(l_{2}R-2)}\right], \end{array}$$

provided this is greater than 0. If *l*_2_*R* < 2, then the population explodes. If *l*_2_*R* > 2, then the critical value of *k*_*e*_, above which hare-hunters can survive is $k_{e}=\frac{l_{3}l_{2}(l_{2}R-2)}{F}.$ Below this value, hare-hunters are driven to extinction and above this value, they converge to the steady state level H_TOT_ given above.

## Supplementary Material

arab125_suppl_Supplementary_Appendix_1

arab125_suppl_Supplementary_Appendix_2

arab125_suppl_Supplementary_Appendix_3

## Data Availability

Analyses reported in this article can be reproduced using the data provided by Ibbotson et al. (2021).
