# Supplementary material for: Dying to cooperate: the role of environmental harshness in human collaboration
Source: Behav Ecol. 2021 Nov 12;33(1):190–201. doi: 10.1093/beheco/arab125 (PMC9113174; doi:10.1093/beheco/arab125)
Supplement: arab125_suppl_Supplementary_Appendix_1 [file arab125_suppl_supplementary_appendix_1.docx]

Appendix 1

Code for all simulations available at:

<https://github.com/crjimene/stag_hare_game/blob/main/stag_hare_model.nlogo>
