# Supplementary material for: Dying to cooperate: the role of environmental harshness in human collaboration
Source: Behav Ecol. 2021 Nov 12;33(1):190–201. doi: 10.1093/beheco/arab125 (PMC9113174; doi:10.1093/beheco/arab125)
Supplement: arab125_suppl_Supplementary_Appendix_2 [file arab125_suppl_supplementary_appendix_2.docx]

Appendix 2


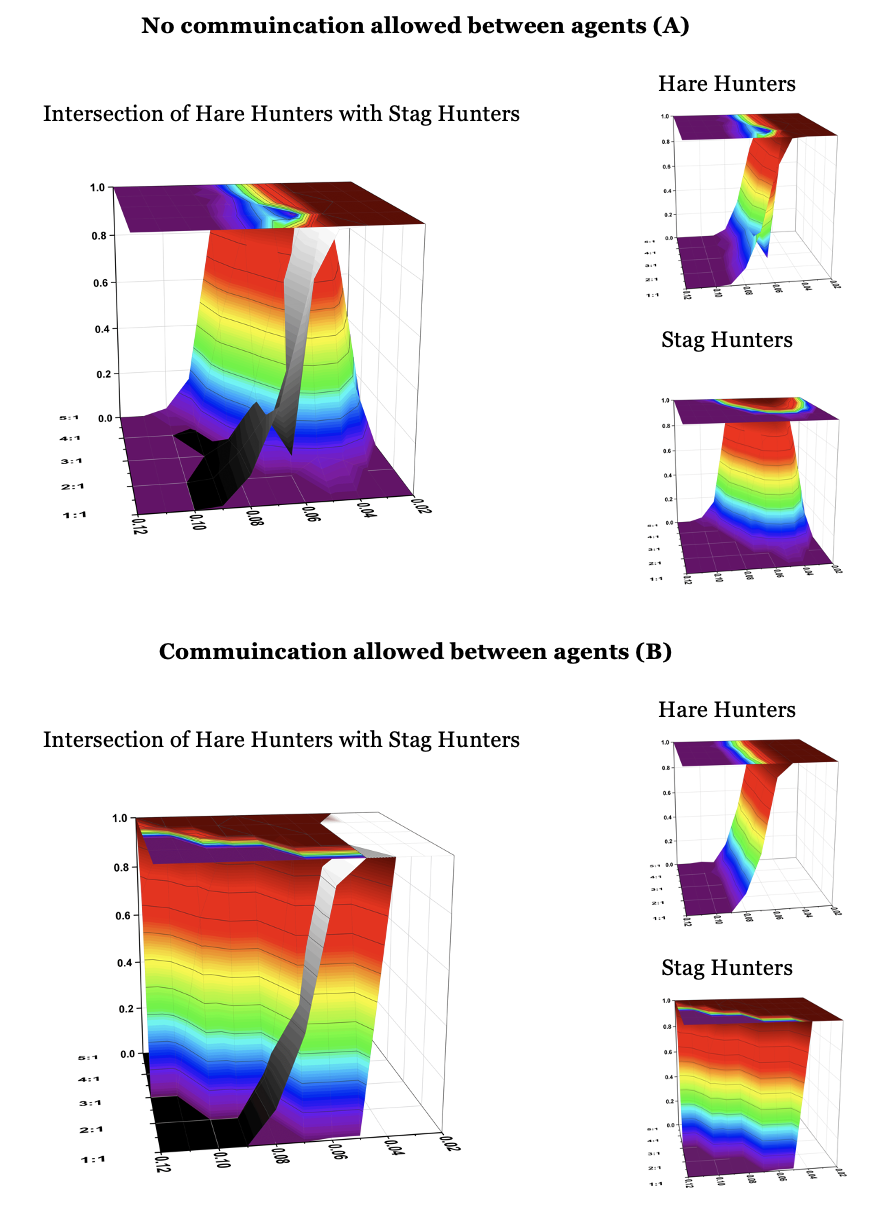


Figure 7. *A 3D representation of the data in Figure 3.* ***(A)*** *No commuincation allowed between agents.* *The survival probability (0-1) of a population being alive after 50,000 units of model time (z) of Hare Hunting and Stag Hunting Strategies as a function of Payoff Ratio (y) and Environmental Harshness (x).* ***(B)*** *Communication allowed between agents.*
