# Supplementary material for: Dying to cooperate: the role of environmental harshness in human collaboration
Source: Behav Ecol. 2021 Nov 12;33(1):190–201. doi: 10.1093/beheco/arab125 (PMC9113174; doi:10.1093/beheco/arab125)
Supplement: arab125_suppl_Supplementary_Appendix_3 [file arab125_suppl_supplementary_appendix_3.docx]

Appendix 3

We had two principal lines of interest in the human data experiment (1) whether harsher environments led to more switching to the high-risk Stag Hunt strategy and (2) whether participants were more likely to switch to the Stag Hunting strategy when energy was low/death was imminent. With respect to (1) Participants never experienced the same energy background decay rate twice. Each participant took part in 6 trials in which the energy decay rate was either 0.02, 0.025, 0.03, 0.035, or 0.04, the order of which was randomised for each participant. So environmental harshness is a between-subjects factor representing one data point/observation per participant. With respect to (2) participants made multiple decisions about whether to switch to stag hunting or switch to hare hunting within their 6 trials so is potentially more of an issue for pseudoreplication. To control for this, we collapsed all within participant data to an average energy when performing a Hare to Stag Switch or a Stag to Hare Switch. Thus one data point per participant per condition as if this comparison was between-subject analysis as in (1). A dependent t-test between the two groups shows a significant difference t(131)=11.49, p<0.001 between the mean energy level of Stag to Hare switch (M=10.68, SD= 3.22) and Hare to Stag Switch (M=7.44, SD = 2.69), Figure 8. So, when we reduce the datapoints to one per participant we find the same pattern of results as when we analysed all data points in the binomial regression, showing a fairly robust effect of energy levels on strategy choice.


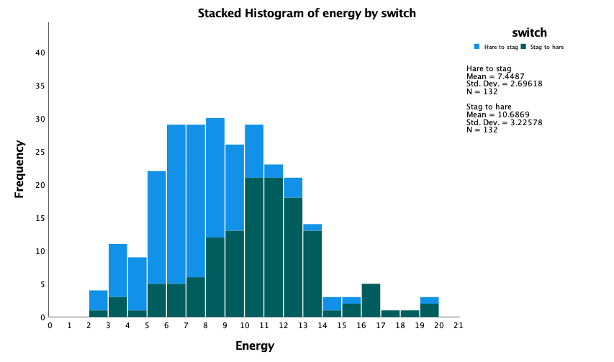


Figure 8. *A histogram of the average, per participant energy level at the time of switch from Hare to Stag (light blue) and from Stag to Hare (dark green).*
